# Are cancer helplines effective in supporting caregivers? A systematic review

**DOI:** 10.1007/s00520-019-04807-z

**Published:** 2019-05-16

**Authors:** Leila Heckel, Natalie L. Heynsbergh, Patricia M. Livingston

**Affiliations:** Faculty of Health, School of Nursing and Midwifery, Deakin University, Geelong, VIC 3220 Australia

**Keywords:** Cancer, Cancer information and support services, Caregiver, Helpline, Oncology, Systematic review

## Abstract

**Purpose:**

The aims of this systematic review were to summarize the profile of caregivers accessing cancer helplines, to evaluate caregiver satisfaction with the helpline service, and to review the evidence base of intervention studies testing the efficacy of community-based cancer helplines in improving caregiver health and well-being.

**Methods:**

Four electronic databases (Medline, CINAHL, PsychINFO, and EMBASE) were systematically searched to identify relevant literature, including all articles published in English until May 2018. Reference lists of accepted papers were reviewed for the inclusion of additional potentially relevant articles, gray literature was excluded.

**Results:**

Forty-five publications met the inclusion criteria for this review. Forty-one papers reported on the proportion of caregivers accessing cancer helplines. Twenty-six studies described demographic and clinical characteristics of caregivers and eight reported on call characteristics. Reasons for contacting the service were stated in 21 studies and caregiver satisfaction with the helpline service was assessed in 12 articles. Fourteen studies investigated specific topics of interest (e.g., prevalence of sleep problems, distress screening, or clinical trial participation). Two randomized controlled trials examined the efficacy of cancer helplines in improving caregiver outcomes, with findings showing interventions to be effective in reducing distress and unmet needs, and in increasing positive adjustment.

**Conclusions:**

There is limited scientific evidence regarding the efficacy of cancer helplines to improve caregivers’ health and well-being. More intervention studies are needed to examine the benefits of cancer helplines to this study population to ensure structured referral pathways can be established.

**Electronic supplementary material:**

The online version of this article (10.1007/s00520-019-04807-z) contains supplementary material, which is available to authorized users.

## Introduction

Informal caregivers are required to provide holistic support to cancer patients throughout the cancer trajectory [1]. This is primarily attributable to changes in the administration of cancer treatments with a shift from cost-intensive inpatient services to outpatient clinics [2]. Secondly, cancer is now recognized as a chronic condition due to increased survivorship rates resulting from advances in diagnostic procedures and therapeutic agents [3]. Subsequently, more people now live with this complex chronic disease which requires ongoing monitoring and care [4].

In their role as informal caregivers, family members and friends take on a multitude of responsibilities and caregiving tasks, however, in many instances, with minimal support and without formal training [5]. During their journey, caregivers experience various unmet needs, significant burden and anxiety [6–9], impacting adversely on their own physical and mental health [10].

Intervention studies have been designed and tested in an effort to support informal caregivers in their caregiving role [11–13]. While these trials showed small to moderate effects, suggesting only limited efficacy on caregiver outcomes, they did produce significant improvements in caregiver burden, coping behavior, quality of life, and self-efficacy [1]. The main drawbacks to supportive care interventions are firstly the costs of having them implemented in the health care setting as most interventions are delivered by qualified professionals. Secondly, the time requirement makes it difficult to have them integrated into caregivers’ busy time schedules, and thirdly, the accessibility with limited availability to those living in non-metropolitan areas [6, 14]. However, caregivers require continuous access to support services in order to obtain adequate knowledge and skills for each phase of the cancer care trajectory. Therefore, it is important to explore other potentially feasible solutions for the delivery of cancer information and support to caregivers, such as the role of community-based support services.

In 1975, the National Cancer Institute in the USA established the first Cancer Information Service (CIS), providing high-quality cancer information via telephone to families affected by cancer, health professionals, and the public [15]. The successful implementation of this service inspired many other community-based cancer organizations to develop similar CIS programs worldwide, and in 1996 the International Cancer Information Service Group (ICISG) was established [16]. At present, the ICISG comprises 30 member countries with a network of approximately 50 cancer information services, its main goal being the provision of standards and resources for the delivery of high-quality cancer information [16]. Cancer Information Services are operated by specially trained and highly qualified staff who provide individualized information, cancer-related emotional and practical support, and referrals to follow-up care [17]. Hours of operation vary across services and individuals are required to self-initiate contact via toll-free numbers or the cost of a local call [15, 17, 18].

While cancer helpline users have reported high levels of satisfaction regarding the delivery of cancer information and support [19, 20], less is known about the actual ability of these telephone services to change caller health and well-being. A review of the literature examining the benefits of cancer helplines for people diagnosed with cancer showed a lack of scientific evidence with only three methodological robust studies evaluating the efficacy of cancer helplines in improving patient outcomes [21]. The studies under review revealed mixed results regarding improvements in self-efficacy and psychological distress ranging from no changes to significant reductions in mood swings and feelings of loneliness in individuals diagnosed with cancer. The authors highlighted not only the need for more rigorous efficacy trials to better understand the value of helplines in delivering information and support to cancer patients and survivors but also the importance of evaluating the benefits of cancer helplines to cancer caregivers, given their substantial involvement in patient care [21].

This systematic review aimed to describe the literature reporting on:

- caregiver access to community-based cancer helplines including the following:
  proportion of caregivers accessing the service
  demographic and clinical characteristics of caregivers
  call characteristics (e.g., patterns of use, source of referral, lengths of calls)
  reasons for calling
- User satisfaction
  level of satisfaction with the helpline service
  acceptability and impact of the helpline service
- The efficacy of community-based cancer helplines in improving psycho-social outcomes of caregivers, e.g.:
  psychological distress, caregiver burden, unmet needs
  self-efficacy, empowerment, involvement in decision-making

## Methods

This systematic review was conducted in accordance with the PRISMA statement [22]. A narrative synthesis approach was used due to the heterogeneity of study designs and the inclusion of descriptive as well as intervention research.

### Inclusion and exclusion criteria

The PICO framework [23] was used to develop a systematic search strategy:

- **P**articipants: Adult caregivers of adult cancer patients (any cancer type or disease stage). Studies only focusing on cancer patients were excluded.
- **I**nterventions: Community-based cancer telephone services focusing on improving caregiver health and well-being; studies including cancer dyads (patient and caregiver together) if caregiver data was reported separately.
- **C**ontrol groups: To fully address the research question, randomized controlled trials, single-arm trials (pre-post trials), and studies reporting on the characteristics of caregivers and their satisfaction with cancer helplines were accepted.
- **O**utcomes: There were no restrictions on the type of outcomes for the efficacy trials but studies should target caregiver needs and well-being.

To allow for comparisons with Clinton-McHarg et al. [21] findings, similar inclusion and exclusion criteria were applied. Articles were excluded if they were not published in English, duplicates, not of original research, not a journal article, or not relevant to cancer caregivers. Studies were excluded if they described a telephone-based service which was (a) not community delivered (e.g., hospital hotline to support patients and their families admitted to this health service); (b) community delivered but: focused on cancer screening/prevention (e.g., smoking quitlines), did not address cancer-related issues (e.g., crisis lines for other diseases), examined the delivery of psychological therapies/services (e.g., cognitive behavioral therapy, psychological counseling services), or provided peer support (e.g., telephone support delivered by another cancer caregiver).

### Search strategy and study selection

Prior to conducting this systematic review, protocol registries PROSPERO, Cochrane, and Joanna Briggs were initially searched and no existing systematic review protocols on this particular topic were found. A systematic and comprehensive search of peer-reviewed literature was conducted in four electronic databases: MEDLINE complete, CINAHL complete, PsychINFO, and EMBASE and included all articles published until 24 May 2018. In addition, snowball searching for citations and references of key articles was performed to identify potentially eligible papers. Gray literature was excluded as it often reports on preliminary findings which are inconsistent with final results; with changes in outcomes from conference abstract to full-length publication in up to 41% of cases [24].

The literature search was conducted using the following key search terms:

Cancer and (caregiver* or carer* or spouse* or partner* or famil* or “significant other*” or caller*) and (“cancer telephone support” or helpline or “help line” or hotline or “hot line” or CIS or “telephone counsel*ing” or “telephone support” or “telephone information service*” or “telephone service*” or “cancer information and support” or “information service*”). These terms were searched within the title, abstract, and keywords. Subject headings (e.g., MeSH terms) for each database were also included in the search.

### Review process

An initial assessment against the inclusion criteria was performed by LH for all identified titles, abstracts, and full-text articles. Studies not meeting inclusion criteria were excluded and full text of potentially relevant manuscripts was subsequently reviewed for eligibility by LH and NH. Discrepancies among reviewers were discussed until 100% agreement was reached.

### Data extraction

Data for publications meeting the inclusion criteria for efficacy studies (randomized controlled trials) and single arm studies were extracted into a coding sheet and included: authors, year of publication, country, participants and sample size, study design, description of the telephone intervention, pre/post assessments, primary/secondary outcome measures, and key results.

Data for publications describing caregiver helpline utilization and satisfaction, and for those reporting on cross-sectional analyses were extracted into a separate coding sheet and included: authors, year of publication, country, sample size (percentage of caregivers contacting the service), characteristics of callers (caregivers), topics discussed/reasons for calling, user satisfaction, impact, and outcome (see Online Resource 1).

### Risk of bias assessment

Randomized controlled trials were assessed for risk of bias against the Cochrane Handbook for Systematic Reviews of Interventions [25] and included random sequence generation, concealment of allocation sequence, blinding of participants/personnel/outcome assessors, missing outcome data, and selective reporting of results. An independent review for study bias was conducted by reviewers (LH, NH) and discrepancies in scores were resolved through discussion.

## Results

### Systematic database search

**Fig. 1:**
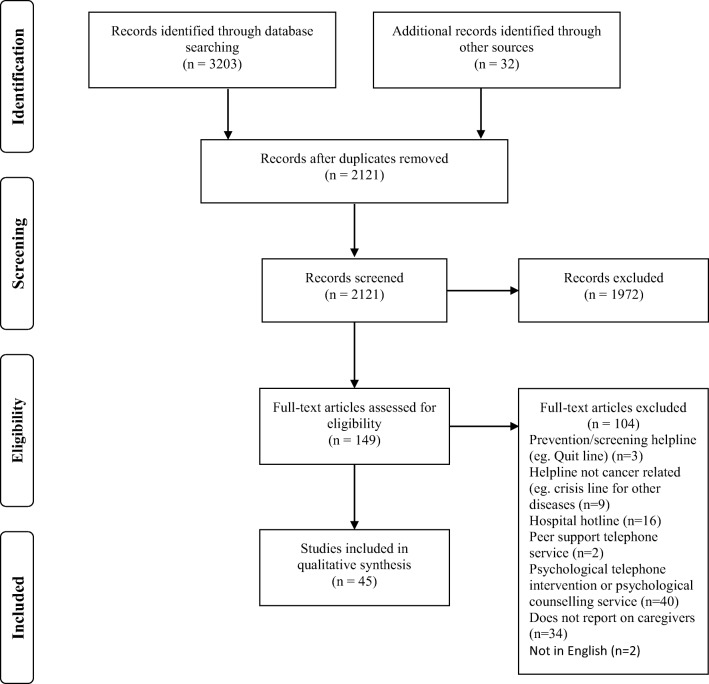
PRISMA flowchart of systematic literature search

The systematic database search yielded a total of 3203 publications, from which 1114 duplicates were removed; 32 additional papers were identified through other sources. After title and abstract screening 151 full-text copies were obtained and screened against inclusion criteria. Of those, 106 manuscripts were excluded as they did not report on caregivers (or failed to present caregiver data separately) or described a telephone service other than CIS. A total of 45 papers were included in this systematic review (Fig. 1).

### Summary of included studies

Table 1 provides an overview of the 45 included research articles. Studies were conducted in the USA (*n* = 19), Australia (*n* = 14), UK (*n* = 7), Netherlands (*n* = 2), Canada (*n* = 1), Nigeria (*n* = 1), and Serbia (*n* = 1). Forty-one papers reported on the proportion of caregivers accessing cancer helplines [20, 26–64, 68], demographic information was outlined in 22 articles [26, 31, 32, 37, 38, 40–42, 44, 46, 49, 51–53, 55–58, 61, 65–67], call characteristics were described in eight studies [30, 38, 41, 43, 44, 52, 61, 66], four papers reported on caregivers’ clinical presentation [32, 38, 43, 66], and reasons for calling the helpline were stated in 21 articles [26, 30, 33, 35, 38, 40–42, 44, 49, 51–55, 58, 61, 62, 64, 66, 68]. Fourteen publications studied caregivers who contacted cancer helplines to answer a specific research question (e.g., prevalence of sleep problems in caregivers) [28–32, 38, 40, 43, 47, 53, 58, 61, 62, 66] and 12 papers informed about caregivers’ level of satisfaction with the helpline service [20, 26, 33, 35, 37, 46, 48, 56, 60, 63, 64, 66]. A comprehensive description of the 42 manuscripts reporting on caregivers’ access to and satisfaction with community-based cancer helplines, including study design and major findings is available for each individual study at Online Resource 1. Two randomized controlled trials presented findings on the efficacy of cancer helpline interventions in improving caregiver outcomes [65, 69], and one single-arm analysis [66], which was part of the two-arm randomized controlled trial [65], reporting on changes in levels of distress and unmet needs as a result of the intervention (both were included in this review, but methods and findings are presented as one study).

**Table 1:** Summary of research articles reporting on caregivers accessing community-based cancer helplines (*n* = 45)

	Number of papers	References	Results
Country of origin	45	20, 26–69	The greatest proportion of studies were conducted in the USA (42%), followed by Australia (31%), UK (16%), Netherlands (4%), Canada (2%), Serbia (2%), Nigeria (2%).
Proportion of caregiver contacts	41	20, 26–64, 68	The overall proportion of caregivers contacting cancer helplines ranged from 14%-67% (call audits - caregivers: 14%-47%; cancer patients: 12%-66%)
Demographic characteristics	22	26, 31, 32, 37, 38, 40–42, 44, 46, 49, 51–53, 55–58, 61, 65–67	The majority of caregivers were: middle-aged (40-60 years), female, Caucasian, well educated, resided in urban areas, the spouse/partner of the cancer patient, lived in the same household with the patient. More than one third came from middle to high socio economic backgrounds, were high income earners, and worked part- or fulltime. The majority were caregivers of patients who were receiving active cancer treatment; the most common cancers enquired about were breast, prostate, colorectal, lung, and melanomas.
Call characteristics	8	30, 38, 41, 43, 44, 52, 61, 66	Most caregivers were first time users, average call duration was 19 min (range: 12-24min). Caregivers most commonly found out about the helpline through health professionals, CIS staff/events, internet or the media.
Clinical characteristics	4	32, 38, 43, 66	One-third of caregivers were found to be distressed or depressed and presented with a mean score of 6 on the Distress Thermometer (range 0-10).
Reasons for calling	21	26, 30, 33, 35, 38, 40–42, 44, 49, 51–55, 58, 61, 62, 64, 66, 68	The most common reasons for calling were to receive emotional/psychological support, to obtain cancer information, to discuss issues related to treatment, symptom management, prevention, diagnosis, and possible causes.
Investigations with a specific research focus (e.g. qualitative or descriptive studies)	14	28–32, 38, 40, 43, 47, 53, 58, 61, 62, 66	Various studies investigated caregivers’ accessing the helpline to answer a specific research question: caregivers living alone compared to general public; caregiver distress, unmet needs, reasons for calling compared to cancer patients; age and gender differences among caregivers; caregivers use of the internet to obtain cancer information; clinical trials discussion by caregivers; distress thermometer administration to caregivers; prevalence of insomnia in caregivers.
Satisfaction	12	20, 26, 33, 35, 37, 46, 48, 56, 60, 63, 64, 66	Caregivers reported high levels of satisfaction (83%-96%): expectations were met or exceeded and CIS staff was rated very positively; caregivers reported increased cancer knowledge, enhanced communication with healthcare teams, and improved decision making.
Efficacy of intervention studies in improving caregiver outcomes	3	65, 66, 69	Two randomized controlled trials focusing on caregiver burden, unmet needs, self-empowerment, distress and post-traumatic growth. Results showed reductions in levels of distress and unmet needs, and an increase in positive adjustment. One single-arm analysis focusing on changes in caregivers’ level of distress/impact of distress on daily life, and unmet needs. Results showed significant reductions for all outcome variables.

### Summary of efficacy studies

#### Risk of bias

Methodological rigor of the two randomized controlled trials [65, 69] was high, with both demonstrating low levels of bias (Table 2).

**Table 2:** Risk of bias assessment for included randomized controlled trials

Authors, year	Random sequence generation	Allocation concealment	Blinding of personnel, participants	Blinding of outcome assessors	Missing outcome data	Selective outcome reporting
Chambers et al. 2014 [69]	L	L	L	L	L	L
Heckel et al. 2018 [65]	L	L	L	L	L	L

#### Study design, content, and efficacy of interventions

Table 3 provides a summary of the two efficacy studies included in the review. The randomized controlled trial conducted by Chambers et al. [69] comprised 354 cancer patients and 336 caregivers who had contacted community-based cancer helplines (Queensland and New South Wales, Australia) for support and had a distress score of ≥ 4 on the distress thermometer (range 0–10). The aim of the study was to compare the efficacy of two telephone-delivered interventions in reducing psychological and cancer-specific distress and improving positive adjustment in both the patient and the caregiver. Outcome measures were assessed at baseline, 3, 6, and 12 months post-recruitment. The two study arms comprised a single session of self-management intervention provided by a helpline nurse and a five-session cognitive behavioral intervention delivered by a psychologist (median session length 46 min, 49 min, respectively). Participants in the brief nurse-led arm received feedback on their distress score, instructions on how to reduce stress, cancer information, psychoeducation, and a resource kit for psychological self-management. The latter contained instructions for managing stress, problem-solving strategies related to cancer issues, educational material to promote a healthy lifestyle, advice on how to reduce the risk of isolation, and an audio CD with relaxation exercises. Participants in the psychologist-led arm received five counseling sessions of psychoeducation, strategies for coping, problem-solving and stress management, cognitive therapy, and the self-management resource kit. Findings related to caregiver outcomes showed a significant reduction in distress and an increase in positive adjustment from baseline to 12 months in both intervention arms. While the estimated sample size of 660 participants at baseline was achieved, the calculated attrition rate of 15% was only attained at the first follow-up assessment point; the problem of high retention rates was noted by the authors. The trial was funded by a grant from beyondblue, Cancer Australia (APP561701), Cancer Council Queensland and New South Wales.

**Table 3:** Characteristics of included randomized controlled studies

First author, year, country	Participants and sample size	Study design	Telephone intervention	Pre/post measures	Outcome measures	Outcomes of interest	Results
Heckel 2018 [65] Australia	Total sample: 432 (216 caregivers and 216 patients with cancer Gender and age: Patient: female (n=122) male (n=94) mean age 59.8 years (Ctrl group), mean age 58.8 years (IN group) Caregiver: female (n=124) male (n=92) mean age 56.3 years (Ctrl group), mean age 57.2 years (IN group) Cancer types: Solid (n=190) Haematological (n=26)	RCT, two arms design Helpline nurse: Three telephone outcalls versus Attention control: Three telephone reminders to self-initiate contact to helpline if needed	Helpline nurse intervention: 4 month duration, including three outcalls to caregivers (at 5-7 days post randomization, 1 months, 4 months). Distress screening and referral if required, discussion of 6 topics addressing caregiver unmet needs. Attention control group: Same duration and number of outcalls to caregivers as helpline nurse intervention, delivered by research staff, reminders to contact helpline service if needed.	Baseline, 1 and 6 months post-intervention	Caregiver burden: Zarit Burden Inventory (ZBI) Depression: Centre of epidemiologic Studies – Depression Scale (CES-D) Unmet needs: Supportive Care Needs Survey –Partner & Caregiver (SCNS – P&C) Supportive Care Needs Survey – Patient (SCNS – Pt) Health Literacy: Health Literacy Questionnaire (HLQ) Self-empowerment: Health education and impact Questionnaire (heiQ)	Primary outcome: caregiver burden Secondary outcomes: (caregiver and patient) Depression Unmet needs Health literacy Self-empowerment Evaluation of helpline nurse intervention	Intervention had no effect on caregiver burden, but significantly reduced caregiver unmet needs from baseline to 1 months post-intervention. No intervention effect on patient outcomes. Intervention was effective in improving self-empowerment in caregivers at risk of depression (having sufficient information to manager their health). Evaluation: Caregivers perceived the helpline service as helpful in reducing their worries (74%), thinking positively about their situation (78%), and in thinking things through (82%).
Heckel 2018 [66] Australia (Secondary analysis of Heckel 2018 [65])	Subsample: 108 caregivers Gender: 46% male 54% female Median age: 59 years (31-77 range) 38% <55 years 32% 55-64 years 30% 65+ Relationship status: 79% spouse, partner Living situation: 83% lived with patient Education: 56% post-secondary education Household size: Median 2 persons (1-8 range) Remoteness: 66% major cities 21% inner regional 12% outer regional 1% remote	Single arm analysis (secondary analysis of a RCT Helpline nurse intervention: Three telephone outcalls	4 month duration, including three outcalls to caregivers (at 5-7 days post- referral, 1 months, and 4 months). Distress screening and discussion of six topics to address unmet needs	Satisfaction survey at 1 months post-intervention	Satisfaction: satisfaction survey Caregiver distress: Distress Thermometer (DT) Unmet needs: Six topics raised for discussion	Caregiver satisfaction with helpline service Changes in caregiver distress and impact of distress on daily activities Changes in caregiver unmet needs (topics discussed)	Satisfaction: 95% stated it was worth their time and effort to take part, 82% stated the program was very relevant to their situation, 96% trusted the information and advice given, 96% stated difficult topics and discussions were handled well, 96% found 13 11 20 nurses well organized, 89% stated that information provided by the nurses was used to assist them in their caregiver role, 91% found referrals and links to community services provided were very relevant to them. Distress/Impact: DT cut-off met: (distress score =>4, impact score =>3): 42% outcall 1 41% outcall 2 19% outcall 3 Levels of distress and impact decreased significantly over time. Topics discussed overall: 82% psychological distress, 45% health literacy, 51% physical health, 44% family support, 28% financial burden, 33% practical difficulties. Caregivers discussing issues related to psychological distress, health literacy, financial, and practical concerns decreased significantly over time.
Chambers 2014, Australia [69]	Total sample: N=690 (354 patients with cancer and 336 caregivers) Gender: Female 83% patient 88% caregiver Cancer types: Breast (31%) Colorectal (9%) Prostate (9%) Hematologic (8%) Lung (8%) Gynecologic (7%)	RCT, two arms design Helpline Nurse: single-session self-management versus Psychologist: delivered 5-sessions CBT	Helpline Nurse intervention: One telephone outcall session involving feedback on DT score and brief instruction in evidence-based strategies to reduce stress + Self-management resource kit. Psychologist intervention: Five telephone CBT sessions, psychoeducation about the psychological impact of cancer, coping and stress management skills, problem solving, cognitive therapy, enhancing support networks + Self-management resource kit.	Baseline, 3, 6, and 12 months post intervention	Psychological distress: Brief Symptom Inventory–18 (BSI-18) Cancer specific Distress: Impact of Events Scale (IES) Post-traumatic growth: The Posttraumatic Growth Inventory (PTGI)	Psychological and cancer specific distress, post-traumatic growth	Psychological distress and cancer-specific distress in caregivers (and patients) decreased over time in both arms. Post-traumatic growth increased over time for caregivers (and patients) in both arms.

The PROTECT multi-center, randomized controlled trial [65] comprised a sample size of 216 cancer patient/caregiver dyads. This study aimed to investigate the efficacy of a nurse-led telephone outcall intervention (*n* = 108) compared to an attention control arm (*n* = 108) in reducing caregiver burden (primary outcome), depression and unmet needs, and to increase self-empowerment (secondary outcomes for both caregivers and patients). Assessment points were at baseline, 1 and 6 months post-intervention. Caregivers in the nurse-led outcall arm received a total of three telephone calls (5–10 days post-randomization, at 1 month, at 4 months) from an oncology nurse at two Australian community-based cancer helplines (South Australia, Victoria) (mean call duration, 22 min). At each outcall, the helpline nurse administered the distress thermometer and offered referral to appropriate services for those with a distress score of ≥ 4 and impact score of ≥ 3. The nurse then raised six topics for discussion to address caregivers’ unmet needs, these included psychological distress, health literacy, caregivers’ own health, practical or financial concerns, or matters related to family life. Caregivers in the attention control group received a total of three outcalls from a researcher at the same time points as those in the nurse-led intervention group (mean call duration, 3 min). Caregivers were given the cancer helpline number to self-initiate contact if needed. Those contacting the helpline would receive the usual support provided by the telephone service, not the tailored intervention. Findings showed that the intervention had no effect on caregiver burden but resulted in a significant reduction in the number of unmet needs from baseline to 1 month in the intervention arm compared to the attention control group. Very few caregivers (6%) in the control arm contacted the helpline service. A subgroup analysis with caregivers at high risk of depression showed a significant intervention effect in the health literacy domain (having sufficient information to manage caregivers’ health). The calculated sample size to be attained by the end of the trial (180 dyads, 90 in each arm) was achieved, however, there were substantial amounts of data lost to follow-up at each post-intervention assessment point which resulted in the study to be underpowered and this was noted as a limitation of the trial. This study was funded by the National Health and Medical Research Council (GNT1044400RM24525).

As part of the randomized controlled trial [65], a single-arm secondary analysis focused on the 108 caregivers in the nurse-led intervention arm [66]. The aim of the analysis was to assess changes in caregivers’ level of distress and impact of distress on daily life activities as measured by the helpline nurse at each of the three outcalls using the distress thermometer, as well as changes in unmet supportive care needs (six topics discussed) over the 4-month intervention period. Findings showed that caregiver levels of distress and impact of distress as well as the frequency of discussions related to psychological distress, health literacy, financial, and practical concerns decreased significantly over time.

#### Acceptability of the helpline intervention

Acceptability of the helpline intervention was assessed through study recruitment rates and engagement with the intervention. Consent rates were not always clearly stated but could be calculated from data reported by the authors. The proportion of eligible callers recruited into the Chambers et al. [69] study was 22%, which was similar to the PROTECT trial [65] which achieved a consent rate of 29%. In terms of engagement, the single nurse-led intervention session in the Chambers’ study [69] achieved a completion rate of 93%, and 53% of participants completed all five of the psychologist-led sessions (median, 4 sessions). Completion rates for the three outcalls in the PROTECT trial [66] ranged from 88 to 95% for participants in the nurse-led outcall arm and from 90 to 94% in the attention control group.

#### Satisfaction with and impact of the helpline intervention

Participants’ self-reported satisfaction and impact of the intervention was only assessed in one of the two randomized controlled trials [65, 66]. Caregivers’ experience with the helpline intervention was very positive. The helpline nurses who delivered the outcalls were viewed as confident and professional, and the information and advice they provided were rated as very beneficial and useful. The majority of caregivers stated that the intervention had a positive impact on their health and well-being, their understanding of cancer, and on developing a positive outlook on life.

## Discussion

This review aimed to assess caregiver access to and satisfaction with community-based cancer helplines and to determine the efficacy of helpline interventions in improving caregiver health and well-being.

### Caregiver profile and level of satisfaction with the helpline service

Studies reporting on call audits showed that the proportion of caregivers accessing cancer helplines ranged from 14 to 47%, which was lower than that reported for cancer patients (12–66%). The majority of caregivers were middle-aged or older, female, Caucasian, highly educated, and married (or in de-facto relationships). The caregiver profile is similar to that reported for diagnosed patients/survivors accessing cancer helplines [21], which highlights the need for more detailed investigations into the reasons as to why caregivers and patients alike, who present with a different profile (e.g., younger, male, less educated) make less use of these services. Studies included in this review suggested the identification of existing barriers for accessing cancer helplines and investigations as to whether their needs are being met by other support services or networks [41]. Fennell et al. [38] reported that the Australian Cancer Council changed the name of their Cancer Council Helpline to “Cancer Council 131120 for Information and Support,” removing the word “help” to increase male usage of this service as evaluations had revealed that users indicated a dislike for asking for help.

Most caregivers were first time users with a call duration of approximately 20 min. Some studies reported that it was common for caregivers to initially request service and cancer information while the need for emotional support emerged later during the call [30, 51], highlighting the need for helpline operators to use probes to detect underlying psychological needs in caregivers when contacting the service at the very first time.

Further, the majority of caregivers contacted the helpline during active cancer treatment, most commonly requested emotional support, information on cancer treatment, and symptom management. Noteworthy, one-third were found to be distressed at the time of calling. It appears that caregivers have high emotional and information needs during the active treatment phase, a time that usually requires high caregiver involvement in the management of patient care in the home setting [1]. Studies have shown that caregivers are not adequately trained for such a complex role and support from the health care system is often lacking [5]. Findings of this review may suggest that cancer helplines have the capacity to compensate for the lack of support for caregivers by off-setting communication barriers between health professionals and families affected by cancer [16].

Caregivers valued the helpline service highly, which was demonstrated by high levels of satisfaction with the quality of information provided as well as with the communication skills of helpline operators who showed high levels of professionalism including sincerity, respect, and empathy, and reports on positive impacts on caregivers’ lives such as enhanced communication with the healthcare team, increased cancer knowledge, and improved decision making. Similarly, the systematic review conducted by Clinton-McHarg et al. [21] revealed high levels of satisfaction and acceptability in cancer patients who contacted cancer helplines for support.

### Efficacy of intervention studies

While findings in the literature overall indicate positive user experiences with cancer helplines, the actual benefits of these services on user outcomes are unclear. The systematic review conducted by Clinton-McHarg et al. [21] which evaluated the benefits of cancer helplines in the cancer patient population only found three intervention studies with strong methodological rigor, and findings of these trials provided limited evidence for the efficacy of helpline services in changing patient outcomes. This review only identified two randomized controlled trials testing the efficacy of cancer helpline interventions to enhance caregivers’ health and well-being. The study by Chambers et al. [69] showed that both, the nurse-led and the psychologist-led intervention were effective in reducing distress and enhancing positive adjustment in caregivers. While the authors highlight the potential benefits of a brief, low-intensity nurse-led intervention; findings need to be interpreted cautiously as the study was conducted without the inclusion of a control group (e.g., no treatment/usual care group) and it is possible that the changes seen in both treatment arms were not entirely generated by the intervention. The PROTECT trial [65] revealed that the nurse-delivered telephone outcall intervention did not demonstrate a significant effect on the primary outcome caregiver burden, and no differences between the two study arms were found for depression and self-empowerment. However, a greater reduction in unmet needs was observed in caregivers receiving the nurse-led intervention compared to those in the attention control group. A secondary analysis [66] also showed significant changes in level of distress and unmet supportive care needs (topics discussed) over the trial period. A possible explanation for these findings may be that the six topics raised by the helpline nurses targeted caregivers’ potential unmet needs which may have caused the significant reduction over the course of the intervention in this domain. In addition, low levels of burden and depression at baseline reported for most caregivers enrolled in the trial may have minimized the chances of detecting any changes over time, as a significant intervention effect in the health literacy domain was only detected in caregivers identified with increased risk of depression at baseline.

### Strengths and weaknesses of efficacy studies

Methodological rigor was high with both randomized controlled trials showing low level of bias. Further, heterogeneity in both trial samples (including both male and female caregivers as well as a wide range of cancer types) allows findings to be applied to the broader cancer caregiver population. However, it is noteworthy that both studies were conducted in Australia, which may limit generalizability of outcomes to other countries due to possible variations in caregiver experiences, cancer helpline operations, and health care systems. The absence of a control group in the Chambers et al. study [69] weakened the evidence for the nurse-led intervention of its potential impact on caregiver health outcomes; however, the brief, one-session intervention in this trial may be more feasible and cost-effective in the helpline setting than the delivery of three outcalls [65].

## Study limitations

This systematic review only included peer-reviewed publications that reported on caregiver access to cancer helplines; it might also be worthwhile to assess the benefits of cancer helplines on medical and allied health care professionals who contact these services to evaluate any impacts on service delivery in these settings. Further, only articles published in English were included in this review, which may have resulted in the exclusion of relevant literature from non-English speaking countries published in national peer-reviewed journals.

## Clinical implications

While limited evidence of the efficacy of cancer helplines in supporting caregivers found in this review impedes evidence-based recommendation, the trials conducted may provide some guidance in improving health care delivery in the cancer population. There is a great need to support caregivers and health professionals can play a crucial role in identifying those in need by screening them for distress and potential unmet needs to allow triage to the most appropriate interventions for those who require support. Creating awareness of cancer helpline services among families affected by cancer is important but self-initiated contacts are less likely to occur [65], therefore structured pathways may need to be established between primary health care providers and cancer helplines to enable telephone outcalls to families who may benefit from some extra help.

## Conclusion

The majority of articles included in this review were descriptive in nature, making it difficult to draw any conclusions about the “true” benefits of helplines in changing caregiver health and well-being outcomes. Only two randomized controlled trials have been undertaken to date, both conducted in Australia, providing limited evidence on the efficacy of helpline services in improving the health and well-being of caregivers. More methodologically, sound intervention studies are needed internationally to strengthen the findings of the trials conducted so far. This will facilitate and guide the establishment of an evidence-based and structured cancer care triage and referral system in the future.

## Electronic supplementary material

Online Resource 1 Cancer helplines and caregivers: Data extraction sheet for the descriptions of cancer caregivers using cancer helplines (PDF 177 kb)

ESM 1 (DOCX 30 kb)
