# Supplementary material for: Are cancer helplines effective in supporting caregivers? A systematic review
Source: Support Care Cancer. 2019 May 16;27(9):3219–31. doi: 10.1007/s00520-019-04807-z (PMC6660576; doi:10.1007/s00520-019-04807-z)
Supplement: Supplementary file 1 — Cancer helplines and caregivers: Data extraction sheet for the descriptions of cancer caregivers using cancer helplines (PDF 177 kb) [file 520_2019_4807_MOESM1_ESM.pdf]

Are cancer helplines effective in supporting caregivers? A systematic review

Leila Heckel[1], Natalie Heynsbergh[1*], Patricia M Livingston[1]

[1] Deakin University, Geelong, Victoria 3220, Faculty of Health, School of Nursing and Midwifery, Australia.

*Corresponding author at: Deakin University, Faculty of Health, Geelong, Victoria 3220, Australia

Tel.: +61 04 3368 5206, email: n.heynsbergh@deakin.edu.au

Online Resource 1 Cancer helplines and caregivers: Data extraction sheet for the descriptions of cancer caregivers using cancer helplines

First author, year, country	Study design and aims	Helpline service, participants and sample size	Characteristics of users/calls	Reasons for calling and topics discussed	Outcome/satisfaction
Boltong 2017 [26]<br>AUS (UK, USA)	Qualitative, descriptive<br><br>To examine the rationale, experiences and outcomes of caregivers and patients who called cancer helpline services in Australia, the UK, and the US	Three cancer helpline services:<br>Cancer Council Victoria CIS (AUS)<br>Cancer Research UK's CIS (UK)<br>American Cancer Society's National CIS (USA)<br><br>Total sample:<br>30 Caregivers and patients with cancer – unrelated, no dyads (10 per helpline service)<br><br>37% caregivers<br>(63% patients)	Gender:<br>73% Female (n=8)<br>27% Male (n=3)<br><br>Age:<br>Mean age: 53 years	Reasons for using helpline:<br><br>Cancer information<br>(not understanding what the cancer diagnosis meant, stunned and confused)<br><br>Not knowing what to do (lost)	Experiences and benefits of using helpline:<br>Questions were answered, helping the caregiver understand their situation, provision of expert knowledge, helpful in providing information, advice and referrals to other services, information and advice at the time when needed<br><br>Impact of using CIS<br>Knowledge as power (confidence and competence in communicating with healthcare team)
Boudioni 1999 [27]<br>UK	Retrospective comparison of cancer incident data and population data with first-time helpline users<br><br>Calls from 1 April 1995 to 31 March 1996	Cancer BACUP Information Service<br><br>Total sample:<br>16 955 first-time callers<br><br>44% relatives/friends<br>(56% patients)	User type:<br>First time users<br><br>Age:<br>Combined data only		
Boudioni 2004 [28]<br>UK	Comparison of helpline caller data and the general population<br><br>Data collection at the time of call over a 5-week period: 13 July 1998–14 August 1998	Cancer BACUP Information Service<br><br>Total sample:<br>552 callers to the helpline<br><br>55% relatives/friends<br>(45% patients)			Compared to the general population:<br>7% of relatives and friends lived alone (20% of patients)<br>Fewer male relatives/friends lived alone<br>89% females over pensionable age living in 2 or more person households

	To examine whether living alone is associated with the use of the helpline service				91% male caregivers (90% female caregivers) lived in 2 or more person households<br>84% male caregivers lived in couple households<br>86% female caregivers lived in couple households<br><br>Differences in enquiries between relatives/friends and patients:<br>6% Hormonal therapy (vs 14% patient)<br>5% Recurrence (vs 10% patient)<br>5% Treatment side effects (vs 13% patient)<br>13% Prognosis (vs 6.8% patient)
Bright 2005 [29]<br>USA	Helpline telephone survey<br><br>To obtain information on callers use of internet to source health information	14 regional helpline services in the US<br><br>Total helpline calls: N=142779<br>33% family/friends<br><br><br>Total telephone survey N=6019<br>41% family/friends<br>(23% patients)			67% family/friends used PC's<br>81% used internet to obtain health information<br>72% used internet to obtain cancer information<br>40% reported finding most information they needed<br>50% reported the information found as very useful, and 43% as somewhat useful<br>34% called the helpline to discuss information found on the internet, and 67% found calling the helpline very helpful, 24% somewhat helpful<br>46% were very interested in email service, 32% somewhat interested

					90% were interested in calling the helpline while on the internet<br>38% were interested in internet moderated chatrooms
Browman 1995 [30]<br>Canada	Census survey of telephone contacts to 6 helpline services in Canada over a 4 day period in April 1992<br><br>Comparison of first-time users with other caller populations. To examine underlying emotional support	Helpline services in the Metro Hamilton district in Canada<br><br>Total contacts:<br>N=946<br><br>Contacts analysed:<br>N=158<br><br>23% (n=36)<br>immediate family<br>12% (n=19)<br>friends, relatives<br>(42% patients)	User type:<br>Of the Spouses, 47% were first time callers<br><br>Of family and friends,<br>44% were first time callers	Reasons for calling:<br><br>Immediate family:<br>61% specific service 22% general information<br>8% emotional support<br><br>Friends/relatives:<br>42% specific service<br>26% general information<br>5% emotional support	First-time callers were more likely to be relatives/friends compared to non-first-time callers
Byrne 2012 [31]<br>USA	Analysis of calls to the helpline over a 3 year period (2006-2008)<br><br>To assess how often clinical trials were discussed by callers<br><br>To assess associations between caller characteristics and the presence of clinical trial discussions	Helpline service in Miami<br><br>Total calls to CIS:<br>N=283,094<br><br>32% family/friends<br>(26% patients)	Family and friends provided care for people diagnosed with the following cancer types:<br>13% Prostate/other urogenital<br>14% Breast<br>20% Colorectal/other gastrointestinal<br>9% Hematologic<br>4% Skin<br>7% Gynecologic<br>14% Lung/other respiratory<br>5% Head and neck<br>3% Melanoma<br><br>Gender:<br>79% female<br>21% male<br>Race/ethnicity:		15.4% family members discussed clinical trials<br><br>Associations between family member characteristics and clinical trials discussions:<br>21% female<br>15% male<br>16% English<br>33% caring for melanoma patients<br>17% non-Hispanic background<br>33% Below 50yrs<br>34% 51-70yrs<br>30% 71+yrs<br>45% College/postgrad education<br>34% Higher income

			96% English<br>80% white<br>89% non-Hispanic<br>Age:<br>25% 40 and younger<br>24% 41–50<br>23% 51–60<br>17% 61–70<br>9% 71–80<br>2% 81 and older<br>Education:<br>7% Less than high school<br>24% High school graduate<br>29% Some college<br>26% College graduate<br>14% Postgraduate schooling<br>Income:<br>12% Less than $20,000<br>16% $20,000–$29,000<br>17% $30,000–$39,000<br>16% $40,000–$59,000<br>23% $60,000–$79,000<br>17% $80,000+		
Chambers 2012 [32]<br>AUS	Cross-sectional survey, baseline data of a randomized controlled trial (telephone intervention). Inbound calls from patients and caregivers to the helpline services<br><br>To assess socio-demographic variables, anxiety, depression, cancer specific distress, unmet needs,	Helpline services in QLD and NSW<br><br>Total sample:<br>N=690<br>(callers with DT>4):<br><br>49% Caregivers<br>(51% Patients)	Caregiver gender:<br>88% Female<br>22% Male<br>Marital status:<br>21% Married/de-facto<br>79% Not married (single, divorced, widowed)<br>Education:<br>34% Non-tertiary<br>66% Tertiary<br>Income:<br>>20000 19%<br>20000-39000: 28%		Comparisons: caregiver vs patient<br><br>Demographics:<br>Caregivers were significantly younger than patients<br>Caregivers were more frequently single than patients who were more likely to be married or in a relationship. Caregivers more often had a tertiary

	reasons for calling, post-traumatic growth<br><br>Comparison between patients and caregivers		40000-59000: 14%<br>60000-79000: 10%<br>80000+: 23%<br>Person provided care for:<br>21% spouse/partner<br>15% parent<br>5% child<br>4% sibling<br>Anxiety, Depression, Somatization:<br>17.7% somatization<br>31.1% depression 35.9% anxiety		education and higher income than patients<br>Distress:<br>Caregivers had significantly higher DT scores than patients when calling the helpline (6.98 vs 6.37 mean) and higher IES intrusion scores (20.65 vs. 16.30 mean)<br>Unmet needs:<br>Mean number of moderate/high unmet needs were higher in caregivers (25.11) than in patients (23.13)<br>Patients reported more frequently<br>Psychological needs, whereas caregivers had more health care<br>services and information needs related to the patient<br>Predictors:<br>Higher intrusion predicted more psychological distress and unmet needs<br>Being single predicted somatization<br>Higher DT score and avoidance predicted depression and anxiety<br>Undergoing treatment predicted anxiety<br>Tertiary education predicted higher unmet needs<br>Being younger and higher avoidance predicted post-traumatic growth

Darrow 1998 [33]<br>USA	Random sample survey of helpline callers in 1996 over a 5-week period<br><br>To evaluate overall satisfaction with call	Total sample:<br>N=2489<br><br>Sub-sample analysed:<br>N=298 (Only those who wanted help coping with cancer<br><br>67% significant others (21% spouse/partner,<br>16% parent, 11% friend/co-worker, 19% other family)<br>(33% patients)		Reasons for calling:<br>Seeking coping information	Satisfaction:<br>83% were satisfied with service<br>65% found helpline staff very knowledgeable<br>77% found helpline staff completely trustworthy<br>62% found printed material very helpful, 76% read all or most of the printed material<br>Impact:<br>98% shared information received with others incl. health professionals<br>42% recommended the helpline to others<br>80% stated information received helped them adjust to the illness<br>85% felt more knowledgeable<br>69% felt more reassured about the situation<br>39% stated the information received helped find support in the community
Davis 2004 [34]<br>USA	Second phase of a randomized controlled trial (pre-post test, two-arm design) conducted at the helpline in the period of March – July 2001<br><br>To assess self-assessed breast cancer risk, breast cancer worries, and breast cancer screening behaviour in callers to the helpline	National Cancer Institute helpline service, Northern California<br><br>Total sample: N=392<br>55% Relative/friends<br>45% general public	Combined data only	Combined data only	Female relatives/friends of cancer patients were more likely to be in mammography maintenance than females of the general public

Davis 1998 [35]<br>USA	Random survey of helpline callers during a 5-week period in 1996<br><br>To assess the satisfaction and impact of treatment and clinical trial information<br><br>Comparison of treatment group with clinical trial group in terms of satisfaction and impact	Total sample: N=2489<br><br>Subsample: N=932<br>Cancer treatment calls: n=579<br>53% family/friends<br>(47% patients)<br><br>Clinical trials calls: n=353<br>54% family/friends<br>(46% patients)		Reasons for calling:<br>Treatment information<br>Clinical trials information	Combined data only for satisfaction and comparison of cancer treatment group vs clinical trials group<br><br>Patients were more likely to report that the information provided was helpful in making a treatment choice than family/friends
Dean 2007 [36]<br>UK	Questionnaire survey of callers using the helpline service during January-March 2007<br><br>To identify the needs and profile of callers and to assess their satisfaction with the service	Breast cancer helpline<br><br>Total survey sample: N=193<br><br>14% Spouse/family/friend<br>(86% patients)	Combined data only	Combined data only	Combined data only
Ekberg 2014 [37]<br>UK	Qualitative interviews with 32 cancer helpline callers between November 2010 and May 2012<br><br>To examine callers' perception of using the helpline service	Five major cancer helplines in the UK<br><br>Total sample: N=32<br><br>31% family/friend<br>(69% patient)	Gender: 80% female 20% male<br>Cancer types:<br>Lung, melanoma, stomach, brain, neck, bile duct, bladder, bowel, mesothelioma, esophageal, kidney		Caregiver perception of helpline service:<br>Positives:<br>Service is anonymous, important resource to gather information, service is filling the support gap<br>Negatives:<br>Call handler lacked empathy, was too formal or blunt, preferred to speak to someone who had gone through similar experience, felt information was delivered in a non-supportive way, service not available during evening hours

Fennell 2016 [38]<br>AUS	Retrospective audit of calls to the helpline between 16 April 2009 and 16 April 2013<br><br>To determine how patients differ from caregivers who call the helpline	Cancer Council Helpline in South Australia<br><br>Total sample: 10940<br>47% caregivers<br>(53% patients)	User type:<br>21.5 % repeat user<br>Gender:<br>83% female<br>17% male<br>Age:<br>53% were 50 and above,<br>47% were aged below 50 years<br>Location/Remoteness:<br>88% resided in major city/inner regional areas<br>12% resided in outer regional/remote/very remote areas<br>Call duration:<br>19.53 min (mean)<br>Levels of distress:<br>Distress level (0-10):<br>6.36 (mean)	Most common topics discussed by caregivers: 44% Emotional, psychosocial issues<br>37% Treatment management 32% Counselling<br>27% Practical issues<br>25% Family, partner issues	Differences between caregivers and patients:<br>Compared to patients, caregivers were more likely to be female<br>below 50 years of age, to call regarding metastasized, widespread, advanced, terminal or unknown stages, to request general information, information on diagnosis, and/or psychological, emotional support<br><br>Compared to caregivers, patients were more likely to be male, over 50 years of age, to call regarding early/localized disease, in the treatment phase, a second primary, in remission, stable or in survivorship phase, to seek advice on treatment and management and/or practical issues<br><br>Caregivers were more likely to find the helpline number via a friend/relative, the media, internet or phone book compared to patients (health professional, cancer Council resource, used number before)<br><br>Caregivers were more distressed than patients

					63% of caregivers required follow-up care (73% of patients)<br>Only 7% of caregiver and 8% of patients received a referral
Freimuth 1993 [39]<br>USA	3 Studies to minimize the knowledge gap between white and African American<br>Study1: Call records to the helpline services to explore characteristics and motivations for information seeking<br>Study2: 54 in-depths interviews with African American callers recruited from 5 helplines<br>Study3: random digit dial survey of 600 African Americans to assess cancer knowledge, attitudes and trust in information sources	Various cancer helplines in the US<br><br>Total sample: (N=10000)<br>50% White<br>50% African Americans<br>Study 1:<br>33% Family/friends<br>(21% white, 12% African American)<br><br>(18% patients - 11% white, 7% African American)	Combined data only	Combined data only	Combined data only
Hawkes 2010 [40]<br>AUS	Consecutive calls to helpline service from patients and caregivers during September-December 2006<br><br>To assess feasibility of routine distress screening and triage by helpline operators	Cancer Council Queensland Helpline service<br><br>Total sample: N=341<br><br>35% Caregivers<br>(65% Cancer patients)	Age, gender, work status:<br>Caregivers were more likely to be younger, female (89%), work casual part time (14%) or be involved in full time home duties (22%), less likely to be unemployed, permanently ill, disabled, unable to work (8%), or retired (21%)<br>Cancer types enquired:<br>Lung (15%)<br>Liver (12%)<br>Brain (9%)<br>Phase/stage of cancer trajectory:	Caregiver's problem list:<br>27% family issues 27% practical problems 71% emotional issues	Caregivers versus patients:<br>Caregivers were 4 times more likely to report a distress level of => 4 than patients<br>Caregivers were more likely to be anxious. Emotional problems were associated with increased distress

			42% were caring for a patient who had undergone surgery in the past 6 months		
Heckel 2017 [41]<br>AUS	Retrospective audit of calls to the cancer helpline by family/friends during the period of January 2010 – December 2012<br><br>To investigate demographic characteristics and psychosocial support needs of family/friends contacting the service	Cancer Council Australia 131120 services (incl. all Australian state and territory helplines)<br><br>Total calls: N=202697<br><br>21% family/friends	User type:<br>84% first contacts<br>16% repeat users<br>Gender:<br>81% female<br>19% male<br>Age:<br>23% < 40 years<br>45% 40-59 years<br>32% 60+<br>How found out about the service:<br>32% Cancer Council (staff, event, educational material)<br>20% media<br>18% internet<br>11% health professionals<br>10% telephone book<br>6% family/friends<br>3% community organization<br>SES status:<br>19% low<br>40% middle<br>41% high	Topics discussed:<br>22% treatment and management 26% psychological, emotional support<br>52% diagnosis, prevention, practical issues	
Heidrich 2003 [42]<br>USA	Structured questions to callers to the North Central cancer helpline to determine feasibility of educational interventions regarding pain and QOL with people calling the service	North Central cancer helpline, located at the University of Wisconsin Comprehensive Cancer Center in Madison<br><br>Total sample: N=306<br><br>Pain substudy (n=205)<br>50% family/friends<br>(50% patients)<br>QoL substudy (n=101)<br>100% family/friends	Mean age:<br>52 years (pain study),<br>50 years (QoL study)<br>Gender:<br>Female<br>85% (pain study)<br>83% (QoL study)<br>Male<br>15% (pain study)<br>17% (QoL study)<br>Phase/stage of cancer trajectory:<br>New diagnosis, no treatment	Reasons for calling: (QoL group)<br>81% feeling anxious<br>71% feeling depressed<br>68% difficulty sleeping<br>63% concerns of how to provide emotional support to patient<br>62% getting information on course and prognosis of disease<br>61% having physical symptoms	Feasibility of conducting telephone-delivered interventions using cancer helpline:<br>Yes responses to question regarding joining future study:<br>89% caregivers in pain study<br>86% of caregivers in QoL study

			20% (pain study)<br>34% (QoL study)<br>New diagnosis, current treatment<br>48% (pain study)<br>37% (QoL study)<br>Post-treatment<br>9% (pain study)<br>8% (QoL study)<br>Recurrence<br>22% (pain study)<br>21% (QoL study)<br>Other<br>1% (pain study)<br>0% (QoL study)	58% worrying how the cancer will affect future plans<br>56% getting information about cancer<br>53% feeling lonely<br>52% feeling angry	
Hughes 2011 [43]<br>AUS	Distress Thermometer (DT) and problem list (PL) use in CIS callers during the period of September – December 2006<br><br>To assess acceptability of DT and PL administration at 13 11 20 service by callers	Cancer Council Queensland helpline service<br><br>Total sample: N=666<br><br>44% Caregivers<br>(56% Patients)	Median call duration:<br>23 minutes<br><br>Median distress rating:<br>(0-10): score of 6<br><br>Problem List:<br>22% practical<br>17% family<br>55% emotional<br>1% spiritual<br>21% treatment<br>12% symptoms<br>21% other		Administration of DT and PL was acceptable with some barriers to overcome for operators.<br>Median call duration was longer when DT + PL was used compared with non-usage (23 vs 10 minutes)<br>Tool used by operators:<br>90% by 6 operators<br>100% by 3 operators<br>74-77% by 3 operators<br>Reasons for not applying the tool:<br>Time or context restrictions, reason for calling was incongruous with DT screening, caller was unreceptive/too ill, communication difficulties, operator forgot to use the tool<br>Callers responses to the tool:<br>94% completed the tool

					6% non-responders<br>Reasons for not responding:<br>43% Unable to describe distress (put a number to it)<br>18% became distressed and were unable to complete the tool<br>Others (not feeling well, called to obtain information material, wanted to get off the phone)
Jefford 2005 [44]<br>AUS	Audit of calls to the CIS over a six year period from 1997 - 2003<br><br>To describe the information and support needs of callers and the response of the service to these needs	Cancer Council Victoria helpline service<br><br>Total contacts: N=42029<br><br>17% family/friends (12% immediate family, 5% extended family)<br>(17% patients)	Age:<br>Immediate family (approximately)<br>35% <30 years 30%<br>30-39 years 20%<br>40-49 years 15%<br>50-59 years 5%<br>60+ years Extended family 35% <30 years 35% 30-39 years 20% 40-49 years 10% 50-59 years 3% 60+ years<br>Mean call duration: 12minutes<br>Cancer type enquired about: Breast cancer	Reasons for calling:<br>Immediate/extended family:<br>70%/80% treatment management<br>50%/50% diagnosis<br>50%/50% psychological emotional support<br>30%/20% recurrence, progression<br>25%/30% practical issues<br>25%/30% prevention, risk factors<br>25/20% side effects<br>25%/30% general information	
Klikovac 2015 [45]<br>Serbia	Call audit of contacts to the national helpline in the period of October 2010 until April 2011<br>To assess caller characteristics and reasons for calling/topics discussed	First national helpline in Serbia, providing psychological support to cancer patients and their families.<br><br>Total sample: N=2748<br><br>25% Family, friends, relatives<br>(66% patients)			

La Porta 2007 [46]<br>USA	National User Survey conducted between November 2003 – March 2004<br><br>10-min telephone survey of first-time helpline users to assess outcomes and satisfaction with the service.	National Cancer Institute's (NCI's) Cancer Information Service (CIS)<br><br>National sample: N=2485<br><br>43% family/friends	User type:<br>100% first time users<br><br><br>Combined data only	Combined data only	Levels of Satisfaction:<br>96% (family/friend) 95% (spouse)<br>very satisfied/satisfied<br>89% (both family/friend, Spouse) expectations were met/exceeded<br>91% (both family/friend and spouse) operator was very knowledgeable/knowledgeable<br>85% (family/friend) 83% (spouse)<br>had significant trust in information provided<br>Impact of helpline contact on knowledge:<br>43% reported significant knowledge increase (36% somewhat)<br>Impact of helpline contact on competence:<br>72% more confident in ability to seek information<br>60% more confident in actively participating in treatment decisions
Lechner 1996 [47]<br>Netherlands	Postal survey to callers of the helpline over a period of 4 weeks in October 1993<br><br>To evaluate the experience and impact of the Dutch national cancer information helpline	Cancer helpline of the Dutch Cancer Society<br><br>Total sample: N=532<br><br>37% family/relatives<br>(46% patients)	Age:<br>Mean age: 43 years		Evaluation of the telephone:<br>Accessibility, approachability, appropriateness, and pleasantness were rated positively with mean scores ranging from 0.94 to 1.45 (from scores -2 to +2)<br>Evaluation of information:<br>Clarity, usefulness, amount, and overall quality were rated positively with mean

					scores ranging from 0.79 to 1.43 (from scores -2 to +2)<br>Evaluation of communication skills:<br>Sincerity, respect, amount of attention, expertise, supportiveness, reassurance, ability to put themselves into the callers situation were rated positively with scores ranging from 0.81 to 1.91 (from scores -2 to +2)<br>Satisfaction:<br>Combined data only<br>Impact on anxiety:<br>Combined data only<br>Impact on expectations:<br>Combined data only<br>Predictors for satisfaction and impact:<br>Best predictor for satisfaction: information received<br>Best predictor for impact on anxiety:<br>Educator's communication<br>Best predictors for impact on expectations met:<br>Information received, communication skills
Linehan 2017 [48]<br>AUS	Inbound calls to the cancer helpline between 12 May – 18 July 2014<br><br>Survey to test the validity of the Distress Thermometer (DT) as a<br>measure of changes in distress after a cancer	Cancer Council South Australia helpline<br><br>Total sample: N=100<br><br>37% family/friends<br>(63% people diagnosed with cancer)		Survey including DT, DASS-21, questions regarding comfort in using DT<br><br>Assessment points:<br>At the start of call<br>At the end of call<br>When completing the survey	DASS-21 scores at time of survey completion:<br>Average anxiety scores (0-3) and depression scores (0-4), both in normal range.<br>Anxiety and depression scores correlated significantly with DT scores<br>Changes in DT scores:

	helpline call, to measure the impact of a helpline call on callers'<br>distress; comfort using the DT, and the frequency of referral to internal support services				A reduction in mean distress scores over time was observed<br>54% reported a recollected score of >= 6 on the DT at time of calling (Time 1) - only 4 of the family/friends received an internal referral for follow up<br>This percentage dropped to 25% at Time two (end of call) and remained the same at Time 3 (survey completion)<br>Comfort completing or administering the DT:<br>81% of family/friends reported feeling comfortable completing the DT<br>In 82% of cases, the nurses were comfortable administering the DT to family/friends
Maibach 1998 [49]<br>USA	Satisfaction survey of a random sample of helpline callers during a 5-week period in 1996, 3 to 6 weeks after initial call<br><br>To assess caller demographics and satisfaction/impact of service regarding prevention and decision making	Cancer helpline service, USA<br><br>Total survey sample: N=2489<br><br>23% family, relatives, friends (77% patients)	Combined data only	Combined data only	Impact:<br>More than 90% of family, friends shared the information received from the helpline with the cancer patient
Manning 2002 [50]<br>Northern Ireland	Retrospective analysis of first-time callers to the cancer helpline over an 18	Cancer information Service (CIS) in Belfast, Northern Ireland	Cancer types enquired by relatives/friends:<br>Breast, Prostate, Lung, Bowel	Topics discussed:<br>20% cancer-related symptoms	

	months period (January 1999 – June 2000)	Total calls: N=571<br><br>46% Relative/friend<br>(21% patients)		30% prostate and testicular problems	
Marcus 1993 [51]<br>USA	RCT of counselling intervention to promote breast screening in female callers to the helpline	Cancer Information Service, USA<br><br>Total sample:<br>N=1831<br><br>57% family, relative<br>(n=1015)<br>27% patients/survivors<br>(n=488)	Combined data only		Combined data only
Marcus 2002 [52]<br>USA	Retrospective audit of calls to the cancer helpline during the period of 1st June 1998 until 30 May 1999<br><br>To describe the caller types and their topics of enquiry	Cancer information and Counselling Line (CICL) of the AMC Cancer Research Centre in Denver, Colorado.<br><br>Total sample: N=1627<br><br>43% Spouse, partner, relative, friends<br>(27% Patients, survivors)	Phase/stage of cancer trajectory:<br>27% newly diagnosed<br>31% in treatment 6% post treatment (survivorship)<br>24% recurrence, palliative care, hospice	Reasons for calling:<br>51% mentioned psychological concerns during the call<br><br>Topics of enquiry:<br>Combined data only	
Meissner 1990 [53]<br>USA	Descriptive analysis of calls to all cancer information services (nationwide sample) in the period of 1983 - 1987<br><br>To examine the information needs of significant others	All cancer information helplines in the US<br><br>Total sample: N=750221<br>25% Significant others<br>(12% patients)	Age:<br>The majority was aged in their 30s and 40s<br>How found out about the helpline:<br>28% health professionals<br>23% written materials, promotions<br>22% telephone book<br>15% other friends, relatives<br>15% TV, radio	Topics discussed:<br>26% Site information<br>13% treatment 12% referral<br>11% counselling<br>10% clinical trials	
Morris 2015 [54]<br>AUS	Descriptive analysis of sleep problems and associated factors in patients and caregivers who called the helpline between 11th	Cancer Council Queensland helpline service<br><br>Total sample: N=734	Gender:<br>84% female<br>16% male<br>Age:<br>18% aged 20-39	Topics discussed:<br>37% practical issues<br>32% psychological, emotional support	Sleep problems:<br>Insomnia Severity Index (ISI)<br>Insomnia mean (0-28): 11<br>Insomnia prevalence:

	December 2012 – 26th March 2013	32% Caregivers<br>(68% Patients)	41% aged 40-59<br>36% aged 60+<br>Education:<br>24% tertiary education (undergrad + postgrad)<br>15% Certificate/diploma<br>54% high school education<br>Phase/stage of cancer trajectory:<br>Time since diagnosis:<br>3 months<br>Cancer types enquired about:<br>13% breast<br>6% male urological<br>11% lower GI<br>7% gynecological<br>8% respiratory<br>10% CNS<br>27% other	14% treatment and management<br>11% general information	63% had a score of 8 or above on the ISI<br>32% had a score of 15 or above on the ISI (moderate to severe insomnia)<br>Distress:<br>Distress Thermometer (DT)<br>DT mean (0-10): 6<br>83% had a score of 4 or above on the DT<br>Caregivers reported significantly higher levels of distress than patients<br>Insomnia and distress:<br>Greater insomnia level was associated with higher levels of distress<br>Caregivers of breast enquiries:<br>37% had moderate/severe insomnia 75% had DT of 4 or above<br>Caregivers of prostate enquiries:<br>14% had moderate/severe insomnia<br>71% had DT of 4 or above<br>Caregivers of other cancer enquiries:<br>31% had moderate/severe insomnia<br>86% had DT of 4 or above
Namboodiri 1993 [55] USA	Analysis of caller data to the helpline regarding tobacco use, mammography, pap test, and clinical trials enquiries during the period of 1986 – 1991.<br><br>To study trends over time	Ohio Cancer information service<br><br>Caregiver data only on clinical trials:<br><br>Over the 5 year period percentage of calls from	Combined data only	Clinical trials	The frequency of enquiries by family/friends regarding clinical trials increased steadily over the five year period

		family/friends regarding clinical trials ranged from 52%-63%			
Reiches 1982 [56]<br>USA	Retrospective audit of calls to the helpline from July 1979 – July 1980	Ohio Cancer Information Service<br><br>Total calls: N=3162<br><br>34% Relatives/friends<br>(14% patients)	Cancer types enquired about:<br>Of those enquiring about:<br>Breast: 26% relatives<br>Colon: 47% relatives<br>Lung: 58% relatives<br>Brain: 72% relatives<br>Leukemia: 61% relatives<br>Hodgkin's disease: 60% relatives	Topics discussed:<br>Of those enquiring about:<br>Causes: 7% relatives<br>Community resources: 34% relatives<br>Screening, diagnosis: 17% relatives<br>Symptoms: 18% relatives<br>Treatment: 57% relatives	
Reubsaet 2006 [57]<br>Netherlands	Cross-sectional survey to evaluate the helpline service and caller satisfaction.<br><br>Callers between December 2003 – March 2004 were invited to complete a postal survey	Dutch national Cancer Information Service<br><br>Total sample: N=1128<br>Surveys returned: N=665<br><br>35% relatives, friends<br>(39% patients)	Age:<br>Mean age: 48.5 years		Satisfaction and impact:<br>Combined data only<br>Predictors for satisfaction and impact (relatives/friends):<br>Best predictor for satisfaction: information received<br>Best predictor for impact on anxiety:<br>Information received<br>Best predictors for impact on expectations met:<br>Information received, communication skills
Rimer 1993 [58]<br>USA	Call audit to the cancer helpline from 1983 to 1990<br><br>To assess demographic characteristics of callers aged 60 and over and to examine relationships between younger and older callers	Cancer information helpline in the US<br><br>Total sample:<br>N=206 104<br><br>22% spouses/relatives<br>(25% patients)	Age:<br>27% aged 40-59 years<br>22% aged 60^{+} years	Combined data only	
Finney Rutten 2006 [59]<br>USA	Call data of family/friends contacting the cancer helpline between 1.	National Cancer Institute's Cancer Information Service (CIS)	Gender:<br>80% female<br>20% male	Reasons for calling:<br>Specific treatment information	Associations between reasons for calling and demographic characteristics:

	September 2002 and 31. August 2003<br><br>To characterize callers information needs<br><br>To summarize differences by demographic characteristics	Total number of calls:<br>N=188 570<br>Subsample analysed:<br>N=83439<br><br>14% Family/friends	Age:<br>39 and below: 27%<br>40-59 years: 47%<br>60+ years: 26%<br>Race:<br>86% white<br>Education:<br>1% Grade school<br>27% Some high school/high school<br>57% college graduate, post-graduate training	General cancer site information<br>Referrals to medical services<br>Support services<br>Psychosocial issues<br>Cancer screening and diagnosis	Gender differences<br>More female than male caregivers inquired about general cancer site information, support services, psychosocial issues, cancer screening/diagnosis, and prevention/risk factors<br>Females more likely to request general cancer site info and to discuss psychosocial issues than males. Males more likely to request specific cancer information.<br><br>Education differences<br>As education levels increased, so did the proportion of calls regarding specific treatment information, referrals to medical services/organizations<br>Frequency of calls about support services where higher in those with less than a high school degree.<br>Caller with higher levels of education more likely to seek specific treatment info, referral to medical services and info about organizations, and less likely to inquire about support services.<br><br>Age differences<br>As age increased, the proportion of calls regarding

					general cancer site information, support services, referrals to medical services decreased. A greater proportion of older caregivers requested specific treatment information than younger caregivers. The percentage of caregivers calling about psychosocial issues was greatest among those aged 40-59. Older age groups more likely to request specific treatment info, younger age groups more likely to request general cancer site info and info on support services
Salako 2016 [60]<br>Nigeria	Descriptive study of call data to three breast cancer helplines over a three months period	Sebeccly Cancer Care & Support Center – three breast cancer helplines in Lagos, Nigeria<br><br>Total number of calls:<br>N=294<br><br>18% Relatives/family<br>(13% patients)	Combined data only	Combined data only	
Slevin 1988 [61]<br>UK	Evaluation of the BACUP service through call record forms and postal questionnaires<br>In the period of October 1985 to November 1987	British Association of Cancer United Patients and their Families and Friends (BACUP) – CIS<br><br>Total number of calls:<br>N=30 000<br><br>39% Relatives/friends<br>(32% patients)	Combined data only	Combined data only	Impact:<br>67% of relatives/friends shared the information received with the patient<br>Overall satisfaction:<br>Combined data only
Sanders Thompson 2008 [62] USA	Audit of call data from African Americans who	National Institute of Cancer CIS service	Combined data only	Topics discussed:<br>10% prevention/risk factors	Spouses, relatives, friends were less likely to seek

	contacted the helpline in the period of 2000-2004<br><br>To assess cancer information seeking among this population group	Total number of calls: N=408 220<br>Calls from African Americans: N=32 834<br><br>30% relatives/friends (26% patients)		47% psychosocial issues	medical referrals, referrals for support services - and were less likely to discuss prevention information but more likely to discuss psychosocial topics
Vanderpool 2011 [63] USA	Cancer helpline call data from 2006-2008 to assess calls received from rural-urban areas regarding clinical trials discussions	National Cancer Institute's CISs<br><br>Total calls received: N=283094<br>Geographically coded calls: N=54842<br><br>34% relatives/friends (27% patients) Urban areas:<br>34% relatives/friends (27% patients)<br>Rural areas:<br>37% relatives/friends (30% patients)	Combined data only	Topics discussed:<br>Clinical treatment trials<br>Total: 16%<br>Urban areas: 16%<br>Rural areas: 14%	Family members/friends are 6 times more likely to engage in a clinical trials conversation than other callers (general public, healthcare providers)
Venn 1996 [64] UK	Callers to the BACUP cancer information service were invited to take part in a postal survey. Study was conducted over 10 working days in August 1991.<br><br>To evaluate access, reasons for calling, satisfaction with the information and service received	The British Association of Cancer United Patients (BACUP) cancer information service<br><br>Total sample who responded to the postal survey: N=282<br><br>62% relatives, friends (36% patients)	Combined data only		Perceptions of the information received:<br>Combined data only<br><br>Impact and overall satisfaction:<br>Combined data only<br><br>Predicting impact and satisfaction:<br>For relatives and friends: Emotional impact was predicted by the nurse's perceived communication skills. Overall satisfaction

					was predicted by the perceived quality of information and the nurse's communication skills
Dewing Ward 1998 [65]<br>USA	A random sample survey of callers to the cancer helpline in 1996 conducted over a 6-weeks period<br>to assess satisfaction and impact of the service	National Cancer Institute's CIS<br><br>Total sample: N=2489<br><br>49% relative, friend<br>(51% patients)	Combined data only	Combined data only	Impact:<br>Sharing of information<br>76% discussed telephone information with the patient<br>76% shared printed material with the patient<br>Satisfaction:<br>Combined data only
Jefford 2005 [68]<br>AUS	Audit of utility and profile of callers to Australian Cancer Council helplines during months of July, August and September 2001-2003	Call data collected by the National Cancer Information Service Network (NCISN), including all Australian states (except Northern Territory)<br><br>Total number of calls: N=76000	Cancer types family/friends enquired about:<br>Colon<br>Prostate<br>Breast Other malignant skin<br>Cervix uteri		
Sutherland 2005 [69] 2005	Calls to the cancer helpline from 1 January 2003 to 31 December 2004 in which callers discussed diet and nutrition	Calls to the Victorian Cancer Council helpline service<br><br>Total number of calls involving diet, nutrition discussions: N=1918<br><br>36% family/friends<br>43% (patients)		Topics discussed:<br>by family/friends<br>13% Unproven cancer-related diet and nutrition cancer<br>9% Risk factors/causes<br>8% Early detection<br>6% Food and nutrition programs<br>4% Cancer prevention<br>Resources provided:<br>Printed material sent to family/friends:<br>41% Tips for eating well<br>7% General nutritional guides, cookbooks<br>5% Healthy eating to reduce cancer risk	
